# Prevalence and European AIDS Clinical Society (EACS) criteria evaluation for proximal renal tubular dysfunction diagnosis in patients under antiretroviral therapy in routine setting

**DOI:** 10.7448/IAS.17.4.19564

**Published:** 2014-11-02

**Authors:** Lorenzo Pitisci, Rémy Demeester, Jean-Claude Legrand

**Affiliations:** Hopital Civil de Charleroi, Belgium Infectious Disease, AIDS Reference Center, Charleroi, Belgium

## Abstract

**Introduction:**

Tenofovir (TDF) is an antiretroviral drug often used in combination regimen in HIV-positive patients. Adverse effects affecting kidneys consist in an increase or a new onset proteinuria, a decrease of glomerular filtration rate (GFR), and/or a proximal renal tubular dysfunction (PRTD) that rarely leads to Fanconi's syndrome. EACS guidelines propose to screen PRTD in patients with chronic renal insufficiency, with a sudden decrease of eGFR, with hypophosphataemia (if non-renal causes such as vitamin D deficiency are excluded) and with a new onset proteinuria. We aim to evaluate the prevalence of PRTD by comparing the group of patients under TDF to the group free of TDF, in our cohort of 300 patients. We also aim to evaluate the accuracy of EACS criteria for screening PRTD in routine settings and to assess the utility of urinary samples in PRTD diagnosis.

**Materials and Methods:**

During two consecutive years, we collected annually blood and urine samples at the same time in our outpatient clinic. We assessed kidney function, plasma levels and fractional excretion of phosphate, uric acid, potassium, plasma glucose and proteinuria. PRTD was defined by the presence of at least two out of the five following criteria: fractional excretion (FE) of phosphate >20% (or >10% when serum phosphate <0.8 mmol/L), non-diabetic glycosuria (positive urine glucose with plasma glucose <70 mg/dL), renal tubular acidosis (urinary pH >5.5 and serum bicarbonate <21 mmol/L), uric acid FE >10% or potassium FE >10%. After the first year, patients with TDF regimen who were diagnosed with PRTD were shifted to TDF-free regimen and included again in the study.

**Results:**

For PRTD (first line), they are expressed in number of diagnoses/total number of patients in this group. The second line resumes the number of PRTD diagnose patients who should have been screened according to EACS criteria.

**Conclusions:**

PRTD screening according to EACS criteria is not sufficient to diagnose every case, especially minor PRTD, mainly because the prevalence is low and its diagnosis remains difficult in routine settings. We recommend performing a urine test including proteinuria every year for patients undergoing TDF treatment. The next step will be to follow PRTD patients to evaluate the time laps until full recovery after TDF shift.

**Table 1:** The table summarises our results

	TDF+ YEAR 1	TDF− YEAR 1	TDF+ YEAR 2	TDF− YEAR 2
PRTD	11/113	2/43	9/91	9/58
Screening accuracy	9/11 (82%)	1/2 (50%)	7/9 (78%)	8/9 (89%)
